# Diverse and unique viruses discovered in the surface water of the East China Sea

**DOI:** 10.1186/s12864-020-06861-y

**Published:** 2020-06-26

**Authors:** Shuang Wu, Liang Zhou, Yifan Zhou, Hongming Wang, Jinzhou Xiao, Shuling Yan, Yongjie Wang

**Affiliations:** 1 College of Food Science and Technology, Shanghai Ocean University, Shanghai, China; 2 Institute of Biochemistry and Molecular Cell Biology, University of Göttingen, Göttingen, Germany; 3 Laboratory for Marine Biology and Biotechnology, Qingdao National Laboratory for Marine Science and Technology, Qingdao, China; 4 Laboratory of Quality and Safety Risk Assessment for Aquatic Products on Storage and Preservation (Shanghai), Ministry of Agriculture, Shanghai, China

**Keywords:** Marine viral community, Surface seawater, Diversity, Archaeal DNA phage, CRISPR, East China Sea

## Abstract

**Background:**

Viruses are the most abundant biological entities on earth and play import roles in marine biogeochemical cycles. Here, viral communities in the surface water of the East China Sea (ECS) were collected from three representative regions of Yangshan Harbor (YSH), Gouqi Island (GQI), and the Yangtze River Estuary (YRE) and explored primarily through epifluorescence microscopy (EM), transmission electron microscopy (TEM), and metagenomics analysis.

**Results:**

The virus-like particles (VLPs) in the surface water of the ECS were measured to be 10^6^ to 10^7^ VLPs/ml. Most of the isolated viral particles possessed a head-and-tail structure, but VLPs with unique morphotypes that had never before been observed in the realm of viruses were also found. The sequences related to known viruses in GenBank accounted for 21.1–22.8% of the viromic datasets from YSH, GQI, and YRE. In total, 1029 viral species were identified in the surface waters of the ECS. Among them, tailed phages turn out to make up the majority of viral communities, however a small number of *Phycodnaviridae* or *Mimiviridae* related sequences were also detected. The diversity of viruses did not appear to be a big difference among these three aquatic environments but their relative abundance was geographically variable. For example, the *Pelagibacter* phage HTVC010P accounted for 50.4% of the identified viral species in GQI, but only 9.1% in YSH and 11.7% in YRE. Sequences, almost identical to those of uncultured marine thaumarchaeal dsDNA viruses and magroviruses that infect Marine Group II Euryarchaeota, were confidently detected in the ECS viromes. The predominant classes of virome ORFs with functional annotations that were found were those involved in viral biogenesis. Virus-host connections, inferred from CRISPR spacer-protospacer mapping, implied newly discovered infection relationships in response to arms race between them.

**Conclusions:**

Together, both identified viruses and unknown viral assemblages observed in this study were indicative of the complex viral community composition found in the ECS. This finding fills a major gap in the dark world of oceanic viruses of China and additionally contributes to the better understanding of global marine viral diversity, composition, and distribution.

## Background

Viruses are the most abundant biological entities on earth and there are over 10^7^ virus particles per milliliter of marine water [1]. Thousands of viral types were predicted by the first viral metagenomic analysis performed in 2002, and at that time, more than 65% of all sequences in the viral metagenome were unknown [2]. More than a decade later, however, a large proportion of unknown sequences remain almost unchanged in environmental viromes [3–8]. This is largely attributed to the fact that commonly used databases, such as SEED [9, 10] and GenBank [11], are dominated by sequences from cultured viral isolates. In addition to their great abundance and high genetic diversity, viruses also possess diversified morphology. Despite being clustered in the same family, some viruses exhibit distinctive morphotypes [12].

Previous virome-based studies have indicated that double-stranded DNA (dsDNA) viruses, especially bacteriophages, comprise the major virioplankton communities in the ocean [13]; even in the Antarctica surface oceanic area, *Caudovirales* comprises up to 72.0% of the total dsDNA virus community [14]; the same case was also found in the North Sea [15] and the Northern Mexico Basin [16]. On a global scale, analysis of the viral diversity obtained from samples collected over 43 voyages of the *Tara* Ocean Expedition indicates that viral communities in the upper ocean are passively transported through oceanic currents and locally shaped by environmental conditions [17]. However, the viromes from coastal, estuarine, and pelagic environments in China have not yet been substantially explored as such. A survey conducted in the Jiulong River Estuary connecting with Xiamen Sea harbor indicates that *Caudovirales* was the major viral group in viromes, and the two most abundant phages were HTVC010P and HMO-2011 [18]. Two other studies focused on viral community polymorphism analysis using *g20* [19] and *psbA* [20] gene targeting for myoviruses and cyanophages, respectively. Recently, metagenomic analysis of the diversity of DNA viruses in the surface of the South China Sea was conducted, providing insight into the viral community in the South China Sea [21]. To date, the diversity and composition of viral community in the surface water of the ECS have yet to be properly documented.

Three places that represent distinctive aquatic environments, Yangshan Harbor (YSH), Yangtze River Estuary (YRE), and Gouqi Island (GQI), were chosen to explore the viral community composition in the ECS. YRE is the entrance of the largest river in China to the ECS, which is a typical mixture of freshwater and marine water. While YSH is frequently and heavily influenced by human activities, GQI is approximately 75 km away from the mainland and is mainly affected by pelagic currents [22]. In previous virome-based research, we explored the single-stranded DNA (ssDNA) viral communities that exist in the surface water of YSH, and found that over 90% of sequences could not be assigned to any known viruses, indicating an unusually broad diversity of ssDNA viruses in the ECS [23].

In this study, we aim to provide insight into the composition of the viral community, mainly dsDNA viruses, in the surface water of the ECS based on genetic analysis of viromes from YSH, YRE and GQI, which span from estuarine to pelagic zones. Additionally, we applied transmission electron microscopy in order to observe viral morphology. We further subjected the viral-affiliated sequences of the viromes to linking to their potential prokaryotic hosts using spacer-protospacer mapping analysis. In addition to the unique viral morphotypes observed in the ECS, the occurrence of putative oceanic archaeal dsDNA viruses was confirmed by genetic analysis. Our results suggested again that the current knowledge of viral features, especially those of archaeal viruses, is merely the tip of the iceberg, and deep exploration will be required to generate in depth understanding of this vast and diverse biological group.

## Results

### Abundance of virus-like particles in the East China Sea

**Fig. 1:**
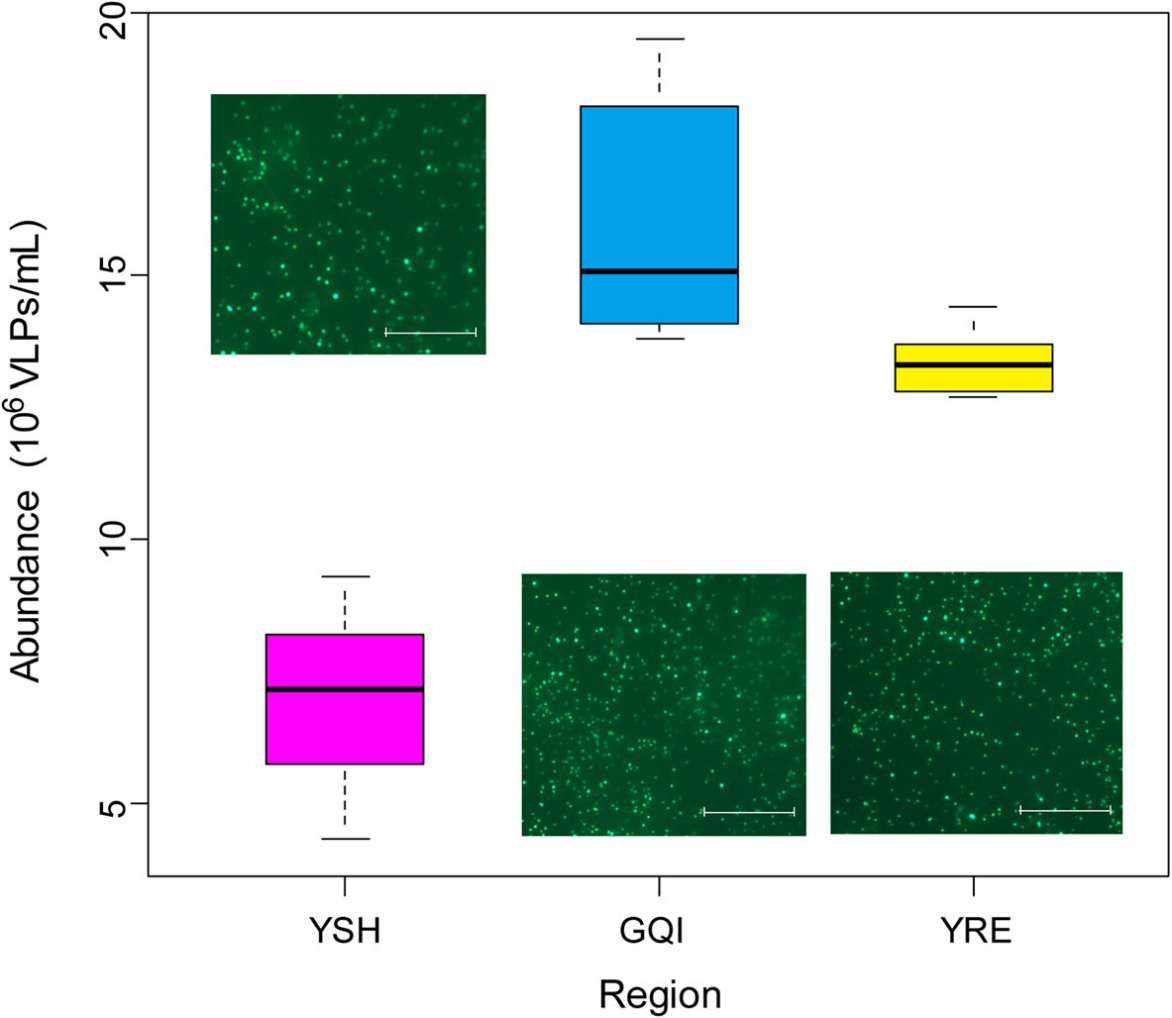
Epifluorescence microscope observation (embedded) and counting of virus-like particles (VLPs) in the ECS. The ordinate indicates the number of VLPs counted per milliliter. The box represents the interquartile range. The horizontal line within the box represents the median. The top and bottom of the box represent the 75th and 25th percentile, respectively. The upper and lower short horizontal lines connecting to the dashed vertical line represent the maximum and minimum, respectively. Scale bar = 10 μm (VLP images). YRE, Yangtze River Estuary. YSH, Yangshan Harbor. GQI, Gouqi Island

Epifluorescence microscopy counting (Fig. 1) showed that the number of VLPs was most abundant in the GQI seawater (1.38–1.95 × 10^7^ VLPs/ml), higher than that of the YRE (1.27–1.44 × 10^7^ VLPs/ml). By contrast, the count for YSH was determined to be 4.32–9.29 × 10^6^ VLPs/ml, which is about half of that found in GQI and YRE. Generally, the offshore surface water of the ECS contained approximately 10^6^–10^7^ VLPs/ml.

### Morphology of the viruses isolated from the East China Sea

**Fig. 2:**
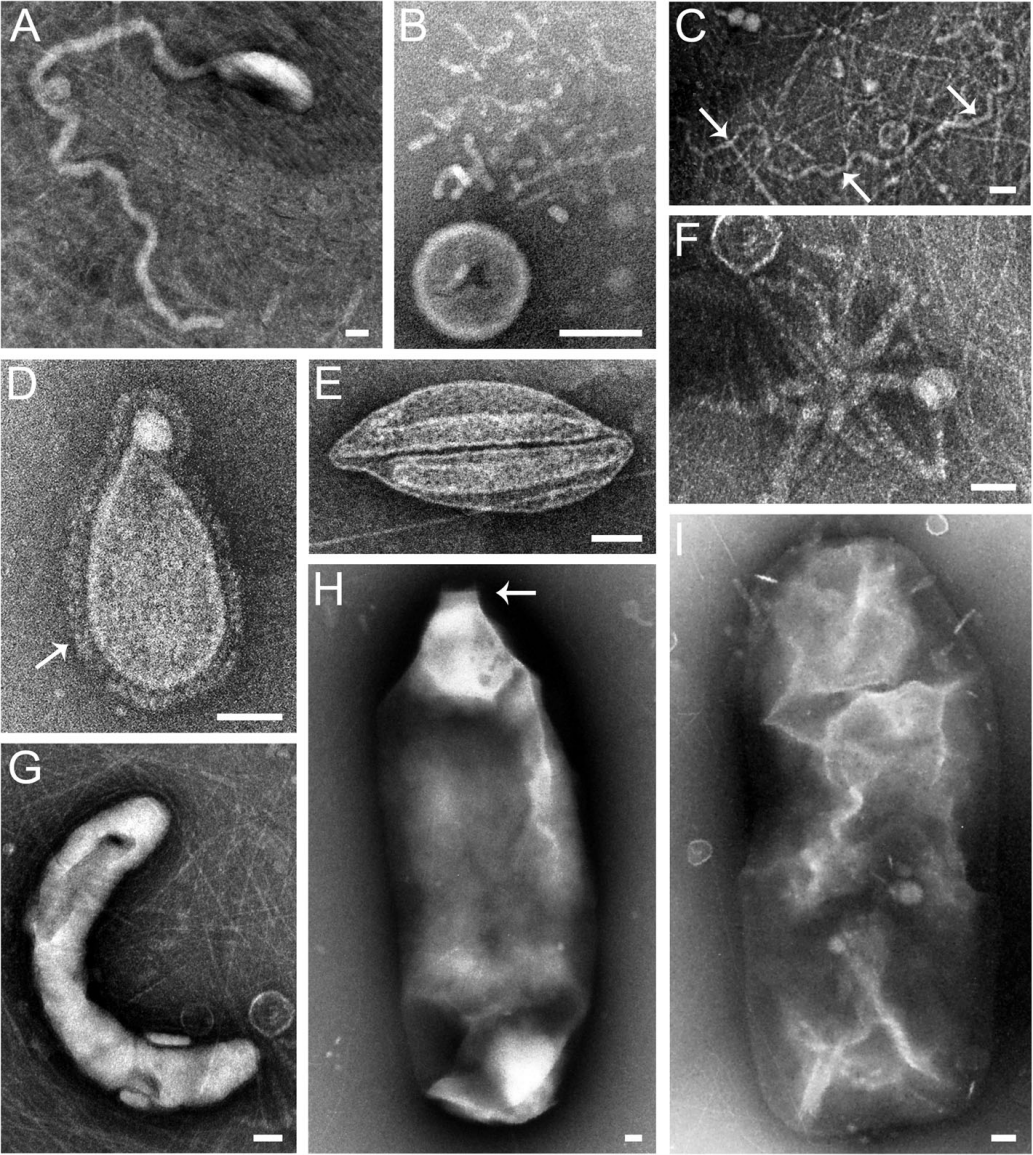
Diverse viruses and virus-like particles in the surface water of the ECS observed under transmission electron microscope. (**a**) atypically elongated head and tail, (**b**) sphere- and rod-, (**c**) long filament-, (**d**) drop earring-, (**e**) lip-, (**f**) starfish-, (**g**) wurst-, (**h**) bottle-, and (**i**) bullet-shaped virus-like particles. White arrows in (**c**), (**d**), and (**h**) indicate the filament virus-like particles, the microcilium of the drop earring-shaped virus-like particles, and the bottle-neck of the giant virus-like particles, respectively. Scale bar = 50 nm for all images

Viruses with a typical structure of head and tail, such as *Siphoviridae*, *Myoviridae*, and *Podoviridae* of *Caudovirales*, were most frequently observed under transmission electron microscope, while viruses possessing an atypical structure with an elongated head (210 × 110 nm) plus a very long tail (1300 × 30 nm) were also found (Fig. 2a). In addition, sphere- (70 nm), rod- (35–40 × 5 nm) (Fig. 2b), and long filament-shaped (1120 × 11 nm) (Fig. 2c) viruses were observed. Most strikingly, a diverse group of unusual morphotypes, e.g., drop earring- (Fig. 2d), lip- (Fig. 2e), starfish- (Fig. 2f), wurst- (Fig. 2g), bottle- (1500 × 560 nm, bottleneck 100 nm) (Fig. 2h), and bullet-shaped (800–1200 × 420–500 nm) (Fig. 2i) virus-like particles was detected in YSH as well. Notably, some of these unusual particles might be virus-like entities, e.g., micro-vesicles, exosomes, or artefacts of the TEM preparation.

### Reads quality control

The results of 454 pyrosequencing for viromes from the YSH, GQI, and YRE, produced 160,393 (average length 525 bp), 151,072 (average length 444 bp), and 62,607 (average length 497 bp) raw reads respectively. After quality control, 118,667 (average length 542 bp), 105,639 (average length 467 bp), and 48,898 (average length 512 bp) reads were obtained for YSH, GQI, and YRE viromes, respectively. With these, we proceeded with downstream analysis. Quality control removed 21.9–30.1% of the total reads.

### Taxonomic composition of the viromes

**Fig. 3:**
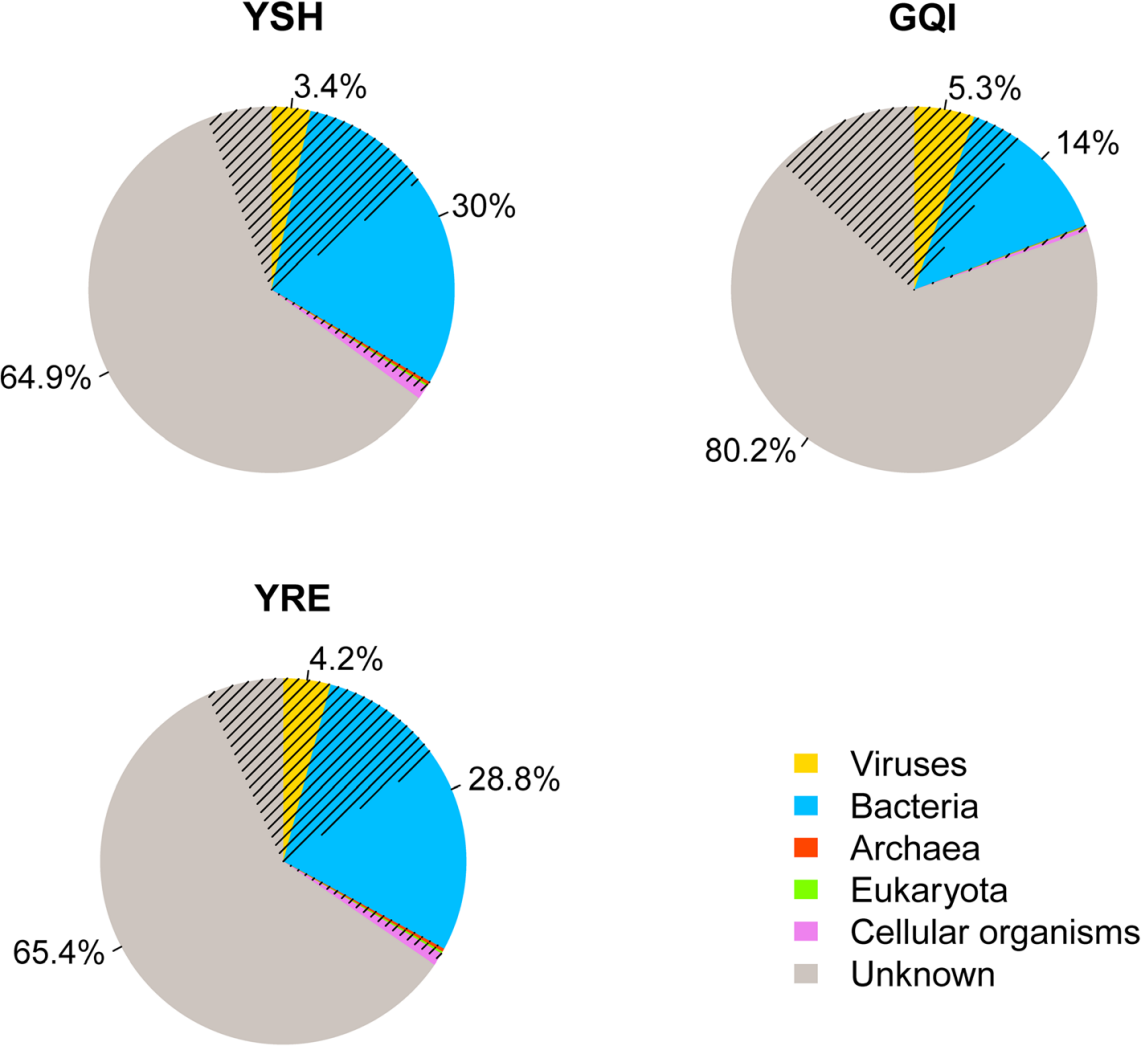
Relative abundance of the virome reads that were classified to different taxonomic groups based on the BLASTx similarity search against the nr database and MEGAN assignment. Reads with no significant hits (thresholds of 1e-3 and 50 on bit score) are defined as “unknown”. Hatched parts represent the portion of reads with significant similarity to the sequences in the local virus database

Only 19.8–34.6% of the reads from the viromes were significantly similar to the sequences deposited in the nr database (Fig. 3). These reads were further classified into viruses, bacteria, archaea, eukaryotes, and cellular organisms (referring to the sequences that were unable to be assigned to bacteria, archaea, or eukaryotes using the MEGAN software). Viral sequences accounted for 3.4–5.3% of the total reads, and bacterial sequences for 14.0–30.0%, while sequences of Archaea and Eukaryota comprised only a small fraction (less than 1%). Most of the sequences (64.9–80.2%) obtained in the viromes were unknown.

To avoid taxon misclassification caused by MEGAN, the second round BLASTx search of reads from the viromes was performed against the locally constructed virus database. As a result, a vast number of reads [YSH, 21.1% (24,983/118,667); YRE, 22.7% (11,097/48,898); GQI, 22.8% (24,069/105,639)] were assigned to the viruses (Fig. 3). Meanwhile, 99.5% of these virus-related sequences matched with predicted viral proteins from the GOV2 dataset, which confirmed the virus-origin of these reads.

Notably, based on the BLASTx search against the GOV2 dataset, 11.5 to 29.3% (YRE, 20.4%, 9990/48,898 reads; YSH, 29.3%, 34,791/118,667 reads; GQI, 11.5%, 12,150/105,639 reads) of these unknown reads (assigned based on the BLASTx search against the nr database) were not identified, indicating diverse and unique viruses present in the ECS, especially in YSH.

**Fig. 4:**
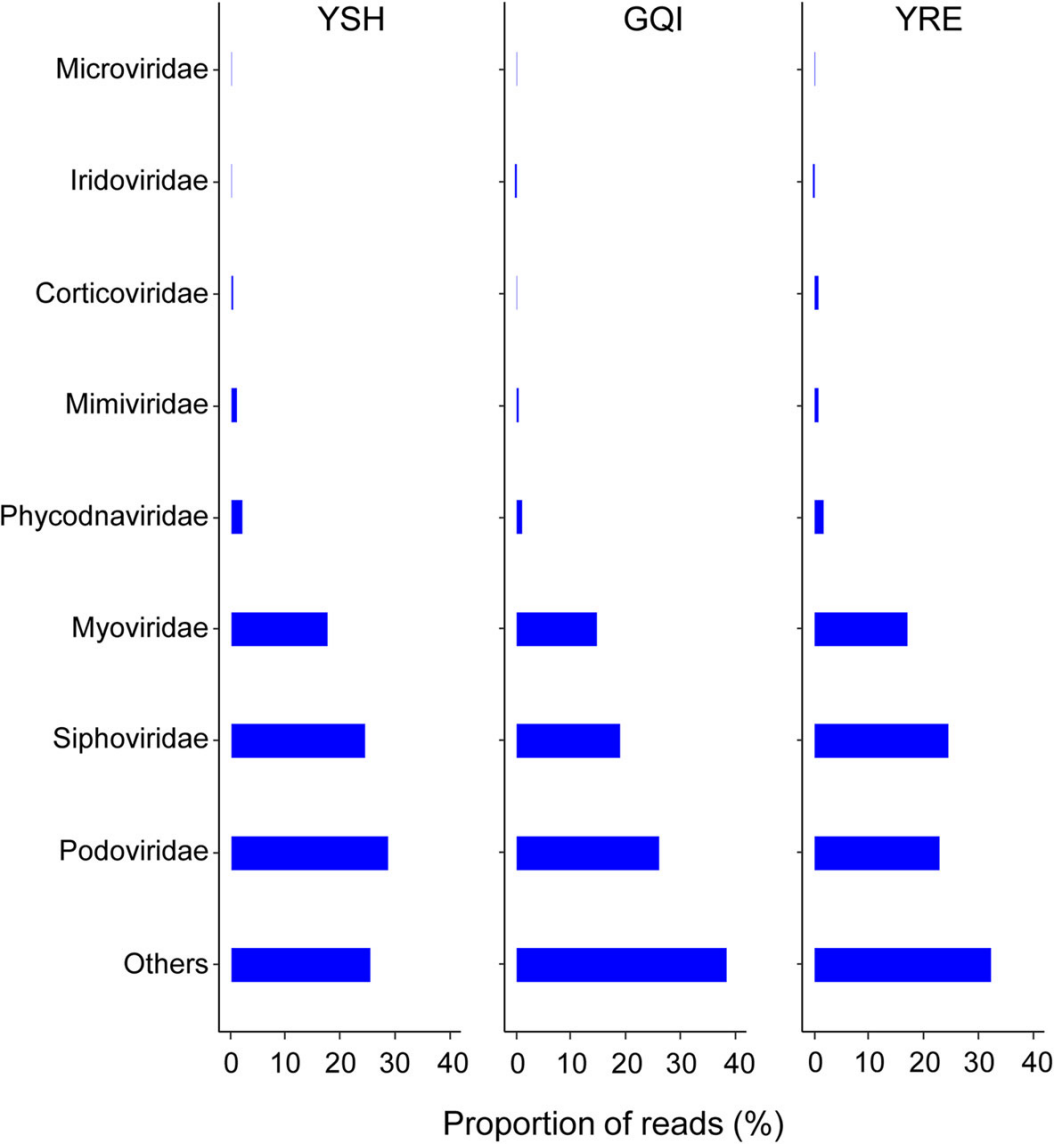
Taxonomic composition of the viromic sequences on the viral family level. Only the relative abundant families that accounted for more than 0.1% are shown. Viral sequences without taxonomy rank are classified as “others”

On the family level, taxonomic compositions revealed both convergence and uniqueness for the three viromes (Fig. 4). Apart from those reads that could not be assigned to known families, most of the reads in these three viromes were classified to the bacteriophage families of *Podoviridae*, *Siphoviridae*, and *Myoviridae*, which belong to the order of *Caudovirales*. Among *Caudovirales*, the *Podoviridae* members were most abundant in the viromes from YSH and GQI, while *Siphoviridae* viruses accounted for the largest proportion in the virome of the YRE. A small number of sequences were grouped to either *Phycodnaviridae*, which infects algae, or the protist-infecting *Mimiviridae*.

**Fig. 5:**
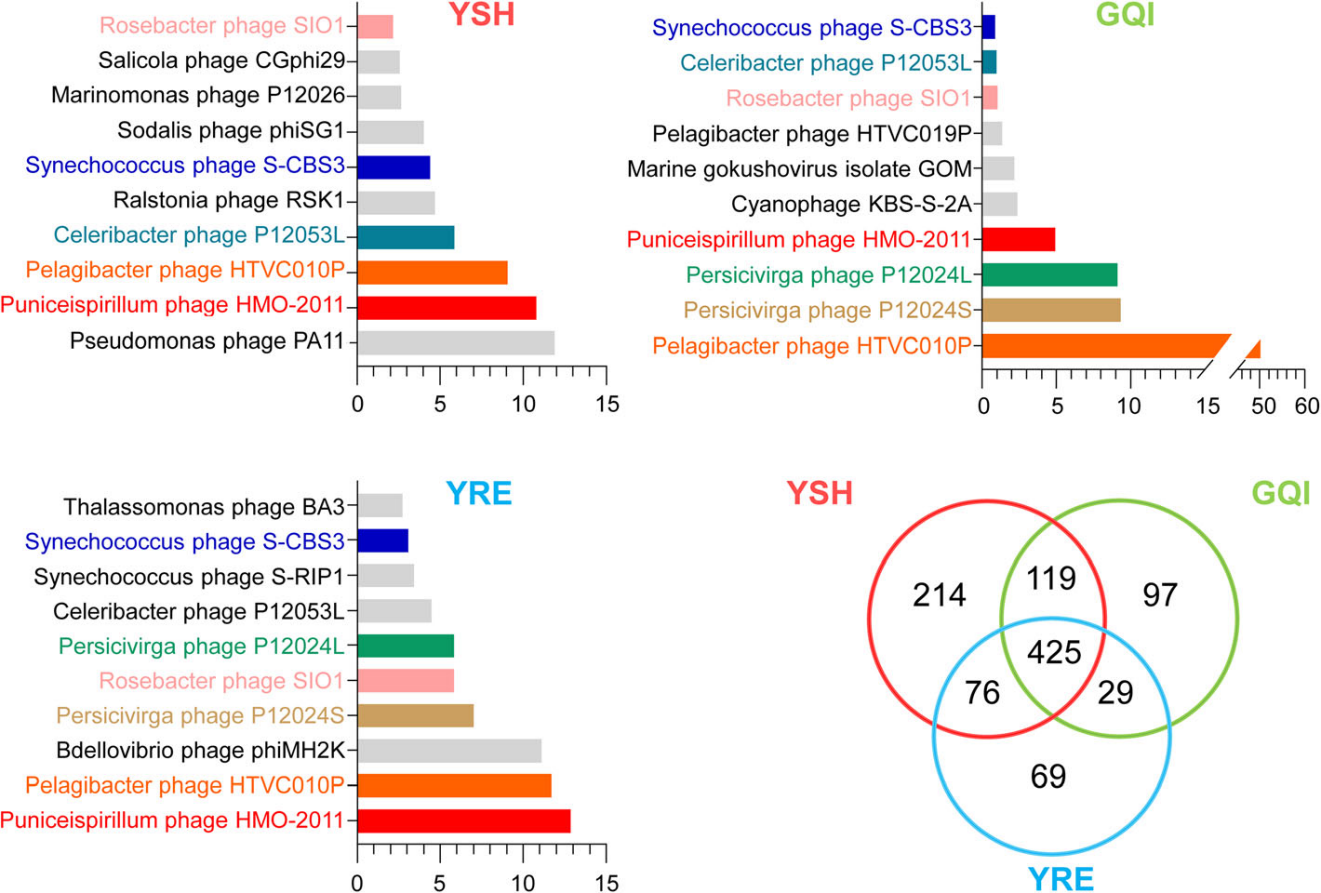
The top 10 most abundant viral species in the three viromes from the ECS. The shared species among ECS viromes are indicated in the same color, and the species specific to each virome are shown in black. The legend of the x axis indicates the proportion of reads of the top 10 species among all the reads assigned to viruses. The number of all shared and distinct viral species among the three viromes is shown in Venn diagram

On the species level, 834, 669, and 599 of the viral species were identified in the viromes from the YSH, GQI and YRE, respectively. Among the three viromes, 425 of the viral species were shared, accounting for 51–71% of the known viral species identified in each virome. This suggests that the majority of known viral species were widely spread in the surface water of the ECS. Meanwhile, 214, 97, and 69 of the viral species were specific to the viromes from YSH, GQI and YRE, respectively (Fig. 5).

### Viral species abundance

The top ten viral species among the identified viral species in each virome were determined by using GAAS (Fig. 5). They all belonged to bacteriophages. Half of these ten viral species, such as *Puniceispirillum* phage HMO-2011, *Pelagibacter* phage HTVC010P, *Synechococcus* phage S-CBS3, *Celeribacter* phage P12053L, and *Roseobacter* phage SIO1, were present in all three viromes; seven were shared between GQI and YRE. However, their relative abundances differed dramatically among the three viromes. For example, the *Pelagibacter* phage HTVC010P accounted for 50.4% of the identified viral species in GQI, but only 9.1% in YSH and 11.7% in YRE.

Given that reads mapping to a single region of a given genome may only indicate the presence of a conserved gene (as opposed to a viral species), the genome coverage of the top 10 viral species was analyzed by calculating the proportion of genes that were mapped by the reads in a given genome [24]. As shown in Table S1, most of the top 10 viral species showed over 70% of the genome coverage. Only a few in the YRE virome ranged from 64.0 to 70.0% (Table S1), which could possibly result from the insufficient sequencing depth because the YRE virome data set contained 48,898 clean reads, only half that of the other two virome datasets. Clearly, the genome coverage analysis confirms the accuracy of the top 10 identified viral species.

### Sequence assembly, ORF prediction, and functional annotation

**Fig. 6:**
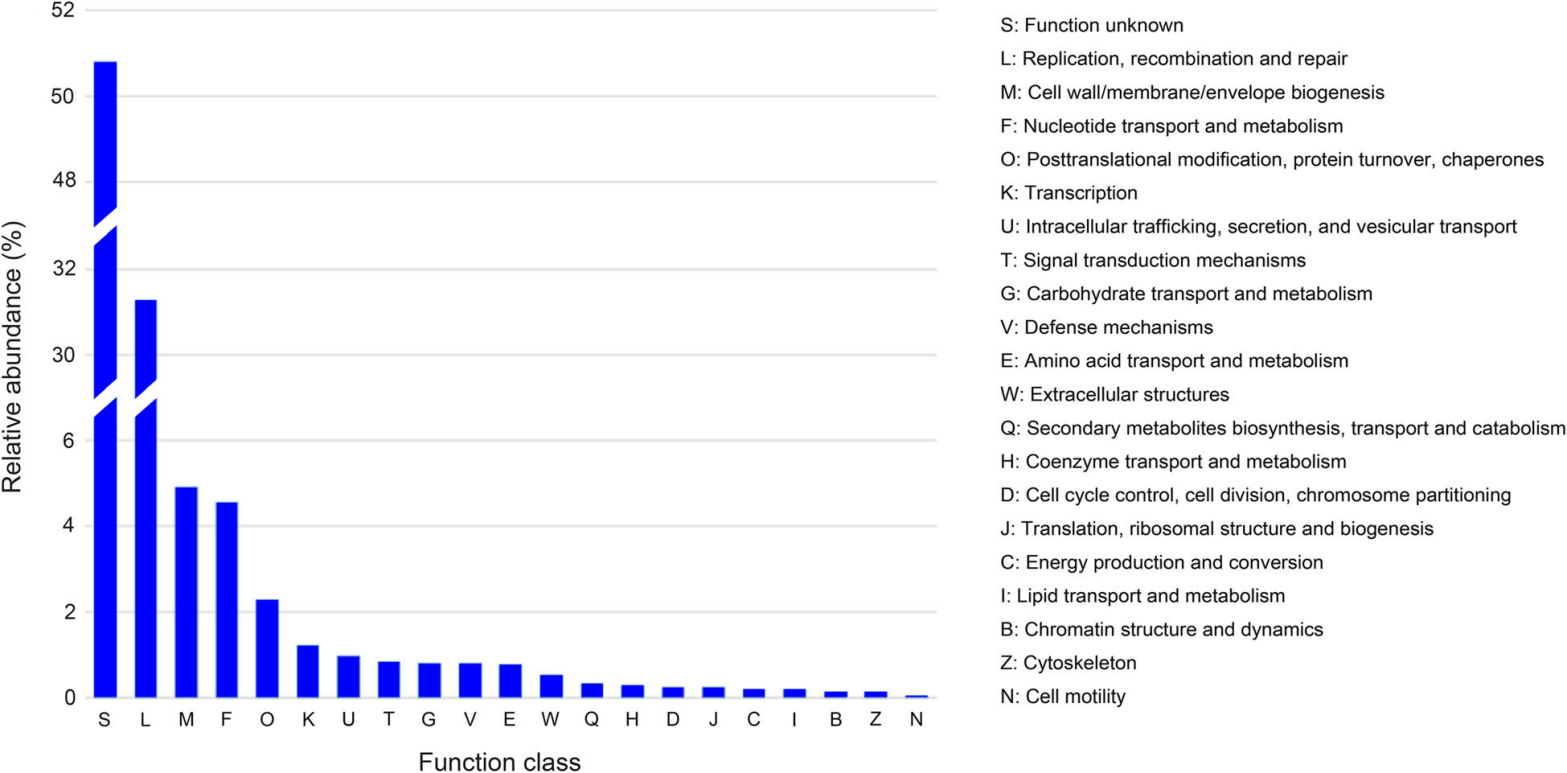
Function classes of the viral ORFs from the ECS viromes

Sequence assembly generated 7443, 9221, and 3984 contigs for the viromes from YSH, GQI, and YRE, respectively. The contig sizes ranged from 107 to 21,309 bp, and the average length was 924 bp (Fig. S1). In total, after ORF prediction and redundancy removal (CD-HIT with parameter set of -c 0.8), 17,789 unique ORFs of over 100 amino acids were retrieved, of which 19.1% (3401) matched known proteins as determined using the eggNOG-mapper, while 26.0% (4632) got hits based on the NCBI Batch CD-Search tool. As for the eggNOG-mapper annotation, 2483 unique ORFs fell into 21 Clusters of Orthologous Group categories (COG Cat.) (Fig. 6 and Table S2). Among these 21 function classes, “S: function unknown” (1261, 50.8%) represented the largest group, followed by “L: replication, recombination and repair” (777, 31.3%), “M: cell wall/membrane/envelope biogenesis” (122, 4.9%), “F: Nucleotide transport and metabolism” (113, 4.6%), and “O: Posttranslational modification, protein turnover, chaperones” (57, 2.3%). The rest were all less than 2%. The annotation details are shown in Table S3 and Table S4.

### Protospacers targeting analysis of the viromes

**Fig. 7:**
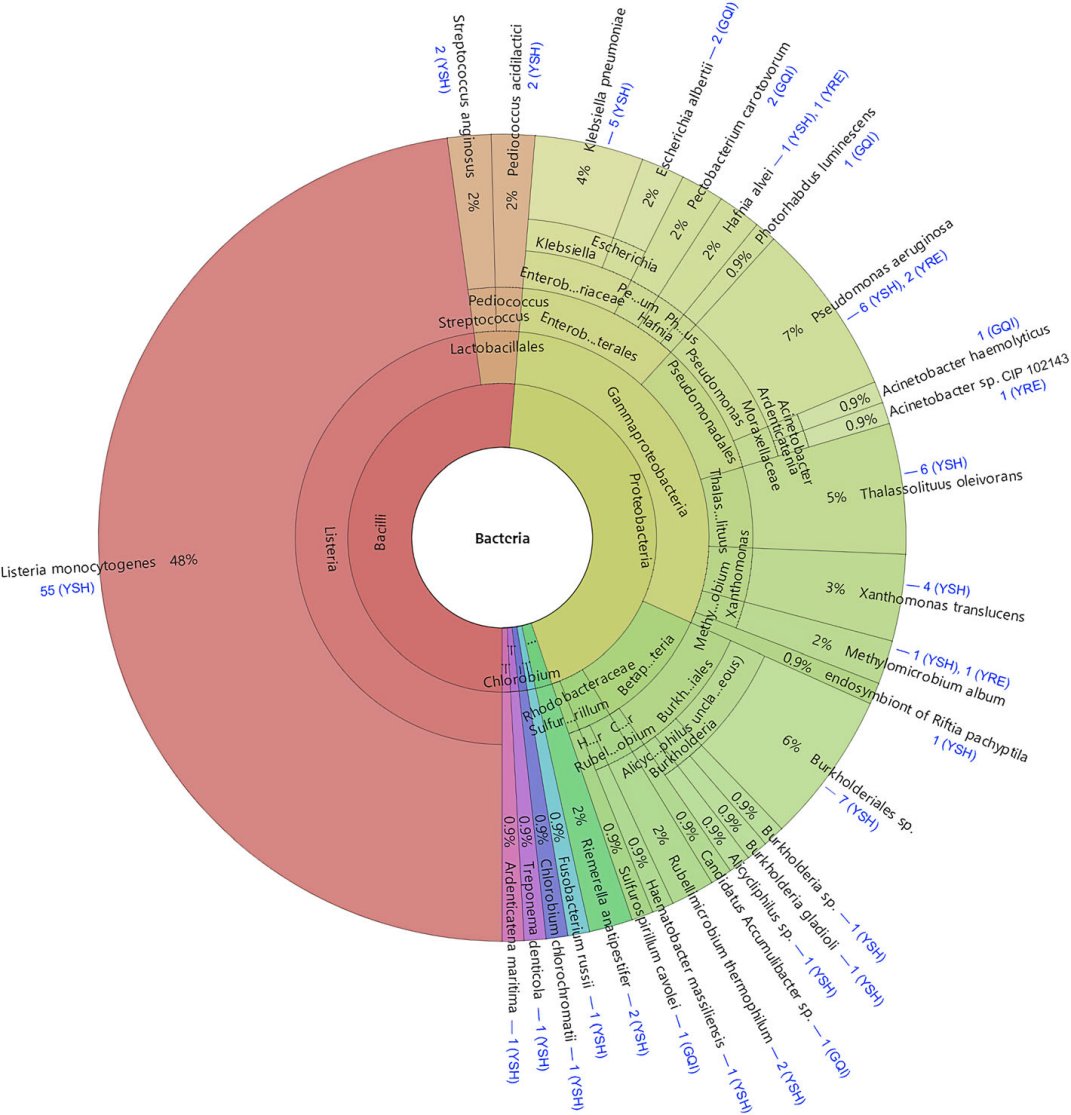
The bacterial source of CRISPR spacers and the number of matched viral sequences in each virome (shown in blue) displayed by Krona [25]. Taxonomy nodes are shown as nested sectors arranged from the top level of Bacteria at the center and progressing outward to the species level

For the three viromes, 115 spacer-protospacer matches were identified (Fig. 7). Seven, five, and 90 spacers were found to be identical to seven, three, and 30 viral sequences in the GQI, YRE and YSH viral metagenomic data sets, respectively (Table S5), revealing one-to-many and many-to-one characteristics. All of the matched protospacer sequences were related to bacterial CRISPR spacers only. Among these matches, the most interesting one was contig_13 from the YSH virome. It was 7480 bp in length and annotated as “viruses”, showing matches with 55 spacers from various *Listeria monocytogenes* isolates (Fig. S2, Table S6). With little doubt, contig_13 can be considered as a partial sequence of an entirely new *Listeria* phage discovered in YSH. Interestingly, an uncultured Mediterranean phage uvMED-like sequence (IFVXWXA02D9OPB, 720 bp) in the YSH virome matched with five spacers of *Klebsiella pneumoniae* and two spacers of *Pseudomonas aeruginosa* (Fig. S3, Table S5), which suggested either a wide host range for this bacteriophage or a conserved region that is present in both *Klebsiella* and *Pseudomonas* phages. A *Vibrio* phage-like sequence (ITRU7KW04IX893, 615 bp) in the YRE virome was linked to *Acinetobacter* sp., which may have suggested a new phage-host relationship. Altogether, we linked 40 viral sequences (9 contigs and 31 unassembled reads) to 28 specific bacterial hosts (Fig. 7, Table S5).

### Uncultured marine thaumarchaeal dsDNA viruses and magrovirus in the ECS viromes

Since the uncultured marine thaumarchaeal dsDNA viruses and magroviruses are the two major groups of archaeal viruses that are widespread in surface water [26, 27], we were intrigued to determine whether or not they were present in the ECS. Only one read (678 bp) mapped exclusively (96% identity) to the genome (118,049 bp, contig_156409) of the Group A magrovirus, and the matched genomic sequence encoded partially an ATP-dependent DNA ligase gene and a phage prohead protease gene (Fig. S4) [27]. In contrast, 171 reads (116–750 bp in length) from the ECS viromes mapped to the putative uncultured marine thaumarchaeal dsDNA virus (38,209 bp) (Fig. S4). These results suggested that the two marine archaeal DNA viruses and/or their close relatives do exist in the surface water of the ECS, but at different abundance.

## Discussion

In this study, three representative viromes were prepared and subjected to metagenomic analysis in order to uncover the genetic diversity of DNA viruses in the surface water of the ECS. The sequences assigned to viruses accounted for 21.1–22.8% of all clean reads from the viromes based on BLASTx search against the locally constructed viral database containing all viral sequences from the nr database. This value increased by 4–5 times (from 3.4–5.3% to 21.1–22.8%) in comparison to the search against the nr database. The difference in assignments likely resulted from the misclassification of MEGAN [28] since the relatively small size of the local viral database and the E-value of 0.001 typically used for the nr database appear not to yield false positive assignments (see the results).

When the virome sequences were first compared to the nr database, the majority of sequences with significant matches were of bacterial, not viral, origin. Whereas in fact, bacterial cells were not observed in the purified and concentrated viruses based on both EM and TEM. Additionally, DNase I was applied to the isolated viruses prior to library construction in order to remove contamination from free cellular DNA. This discrepancy likely results from one of several factors: 1) the prophage sequences on the bacterial genomic sequences were falsely annotated to bacteria; 2) some of the bacterial sequences originating from viruses were due to horizontal gene transfer; 3) the presence of auxiliary metabolic genes shared between viruses and their hosts, for example, both cyanophages and cyanobacteria encode *psbA* genes [29]; 4) the DNase I resulted in incomplete removal of cellular DNA [30]. Bacterial genomes are far larger than those of viruses, and can yield a significantly higher number of bacterial sequences in the virome data sets, even if the viromes may have been contaminated only with a very tiny amount of bacterial DNA.

The first metagenomic analysis of the viral community in surface seawater from Scripps Pier and the channel side of Fiesta Island in Mission Bay showed that the viral hits all belonged to the major families of dsDNA tailed phages and some algal viruses [2]. Our results also indicated the dsDNA tailed phages were the most abundant known viruses in the viromes of YSH, GQI and YRE, together with some large algal viruses belonging to *Phycodnaviridae* and some giant viruses belonging to *Mimiviridae*. These results are in accordance with the fact that *Caudovirales* are the dominant known viruses in the viromes compared to eukaryotic DNA viruses [31]. Notably, since *Caudovirales* dominates the available phage sequences in the database by far, the probability of matching members of this order is higher than that of matching to any other phages. It is not surprising that the sequences were dominated by phages in general. Coincidently, viral particle sizes resembling those of giant viruses were clearly observed in the ECS viromes (Fig. 2h and i), while potential viral parasites (virophages) of giant viruses [32] were not detected in the same viromes. Interestingly, our previous work [33–35] showed that virophages were widely distributed all over the world, including diverse ecological environments like the ocean. Whether the lack of virophages-related sequences in the ECS viromes resulted from insufficient sequencing depth, viral isolation methods, or other unknown sources requires further investigation.

Scripps Pier and the channel side of Fiesta Island contained an abundance of the *Roseobacter* phage SIO1 [36]. However, this viral species is not the most abundant one in the ECS, but it is one of the top 10 most abundant viral species and accounts for 1.1–5.9% of the ECS virome reads assigned to viruses. Interestingly, the most abundant viral species in surface water from the Jiulong River Estuary in the southeast coast of China are the *Pelagibacter* phage HTVC010P, the *Puniceispirillum* phage HMO-2011, and the *Thalassomonas* phage BA3 [18], which were all present in the YRE and showed similar patterns of abundance. In addition, the unclassified viral sequences in the GOV2 data sets matched with both virus-related and approximately 70–90% of unknown reads in the ECS viromes. These results imply that similarity of viral communities can be found in different oceanic regions but that their abundance differs amongst these regions, supporting the idea that viruses are widely dispersed and that local environmental conditions enrich for certain viral types through selective pressure [4].

The analysis of the viral community composition demonstrated 425 common species shared in YSH, GQI and YRE, including five of the top-ten viral species, which coincided with the fact that these three sampling regions are geographically connected, especially influenced by water diluted by the Yangtze River and Kuroshio Current (https://earth.nullschool.net/). However, there was a difference in the number of VLPs and of unique viral species in each region, which likely reflected the differences in ecological habitats of these three aquatic regions. For example, GQI is far from the mainland and is exposed to very limited pollution from human activities. Consequently, it maintains primary productivity that is relatively high, especially higher in the availability of microbial hosts, and thus is characterized by the highest number of VLPs. Note that the abundance of the *Pelagibacter* phage in GQI was also consistent with the wide distribution of its host—*Pelagibacter ubique* that is a ubiquitous and predominant marine bacterium found in pelagic environments [37, 38]. In contrast, YSH, the most unfavorable ecological environment among these three sampling regions, is buckling under perennial urban sewage and oil pollution from numerous international ocean-going cargo carriers. It is probable that this results in a more vulnerable and variable ecosystem, plausibly explaining why it contained the lowest number of VLPs but the most diverse viral species [39].

Surprisingly, in this study, we observed the unusual morphotypes of the drop earring-, lip-, starfish-, and wurst-shaped virus-like particles. Most of them had not yet been observed in other environments. Interestingly, the starfish-shaped virus-like particles resembled the Aster-Like Nanoparticles (ALNs) discovered in pelagic environments, especially in meso- and eutrophic waters [40]. The ALNs were not confidently considered as viruses since DNA was not detected in them. However, it remains unknown whether or not the ALNs contain RNA. These exotic virus-like particles are reminiscent of unique archaea-specific viruses [41] and whether these are bona fide archaeal viruses is worthy of further investigation. The atypical head-tailed structure of virus shown in Fig. 2a resembles the magroviruses that infect Marine Group II Euryarchaeota since magroviruses are considered to possess the head-tail virion structure [27] and, importantly, were also detected in the ECS viromes. Meanwhile, the putative marine thaumarchaeal viruses also appear to be present in the ECS. Coincidently, they were discovered in the surface (10 m) and oxygen-starved basin waters (200 m) of the Saanich Inlet and globally in ocean surface waters as well [26]. These results provided genetic evidence, once again, for the common presence of this thaumarchaeal virus and magroviruses in the global ocean [27]. Finally, the bottle- and bullet-shaped virus-like particles (Fig. 2h and i) resembled the protozoa-infecting giant viruses, such as pithovirus [42, 43] and pandoravirus [44], in both morphology and size; meanwhile, sequences similar to giant viruses were present in the ECS viromes as well. A recent study reported that *Pandoravirus salinus* was repeatedly detected in vessel ballast water viromes from both Mexico and Saudi Arabia [45]. The fact that great ballast water is exchanged worldwide enables archaeal viruses and giant viruses, together with bacteriophages, to spread globally.

The high percentage of uncharacterized ORFs (eggNOG-mapper, 80.9% and Batch CD-Search, 74.0%) also mirrors the diversity and novelty of the ECS viromes. With the exception of class S, the three abundant classes of L, M, and F of eggNOG-mapper annotations all seem to reasonably contribute to viral biogenesis, supporting again the validity of our viral sample collection and treatment. Surprisingly, among the CDD annotations we found Cas4 hits (Cas proteins contributing to the adaptation stage [46, 47]) (Table S3), which demonstrated another example of the exchange of components between CRISPR-Cas (clustered regularly interspaced palindromic repeats and CRISPR associated proteins) systems and mobile genetic elements (MGE) [46].

The CRISPR-Cas system, the adaptive immune system of prokaryotes, functions as specific immune memory (spacers) against foreign genetic materials through integrating fragments of invading nucleic acids (protospacers) into CRISPR arrays via the Cas adaptation machinery [48–50]. In the present study, we performed CRISPR spacer-protospacer matching analysis for all three viromes in an attempt to establish connection between viral sequences and potential prokaryotic hosts. Notably, it has been confirmed that, for phages matching a single CRISPR spacer but allowing two mismatches, the predictions are correct at a rate of 74% on the species level [51]. Therefore, we established, with confidence, the connections linking 40 unique viral sequences to their potential specific hosts (at least 95% identity and 95% coverage).

One dramatic finding points to the link between contig_13 (YSH) and *L. monocytogenes* (55 spacers). As is one of the most virulent foodborne pathogens [52], *L. monocytogenes* and the appearance of its potential phage in YSH indicated the presence of contamination from human domestic sewage in this sea area to a certain extent. To our surprise, the uncultured Mediterranean phage uvMED-like sequence (YSH) was linked to the two bacterial species *K. pneumoniae* (5 spacers) and *P. aeruginosa* (2 spacers). Even more surprisingly, the targeting sites for all of these spacers (55 + 5 + 2) were located in the viral DNA methylase gene, e.g., Cytosine-C5 specific DNA methylase for contig_13_ORF15 (YSH) and DNA N-6-adenine-methyltransferase for the uncultured Mediterranean phage uvMED-like read_IFVXWXA02D9OPB_ORF1 (YSH) (Fig. S2, Fig. S3 and Table S6). This spontaneously gave rise to the thought that, at the very beginning of the prokaryotic viral defensive process, upon the injection of viral DNA, the CRISPR-Cas machine specifically recognizes and cuts down the viral DNA methylase gene. Consequently, it may impair the production of infectious virus [53] and/or result in the inability to protect phage DNA against restriction-modification upon the host infection [54]. This scenario deserves to be explored systematically in order to uncover far more unknown and intriguing stories surrounding CRISPR-Cas defense mechanisms. Additionally, it also suggested that prokaryotes take advantage of two types of anti-phage defense mechanisms simultaneously, including restriction-modification and CRISPR-Cas systems, to resist the evolutionary pressure imposed by phage predation [55, 56].

Obviously, we cannot completely rule out the possibility that two or more matched viral sequences actually belong to a single bacteriophage species. Furthermore, the absence of matched spacers in archaea may be due to the limited number of known archaeal genomes available thus far. Nevertheless, and more promisingly, the discovery of protospacers in the ECS viral metagenomes certainly strengthens the link of certain kind of viruses directly to a specific host [57].

## Conclusions

In conclusion, this study is the first to investigate the diversity and community composition of viruses in the ECS, which prominently demonstrated the novelty of the viromes that still await comprehensive exploration and analysis, which is necessary to glean insightful knowledge for this aquatic area. It will be particularly intriguing to more deeply explore the exquisitely designed viruses and the new virus-host relationships being discovered in the global oceans, especially in the oceanic areas that have yet to be studied.

## Methods

### Sample collection

**Fig. 8:**
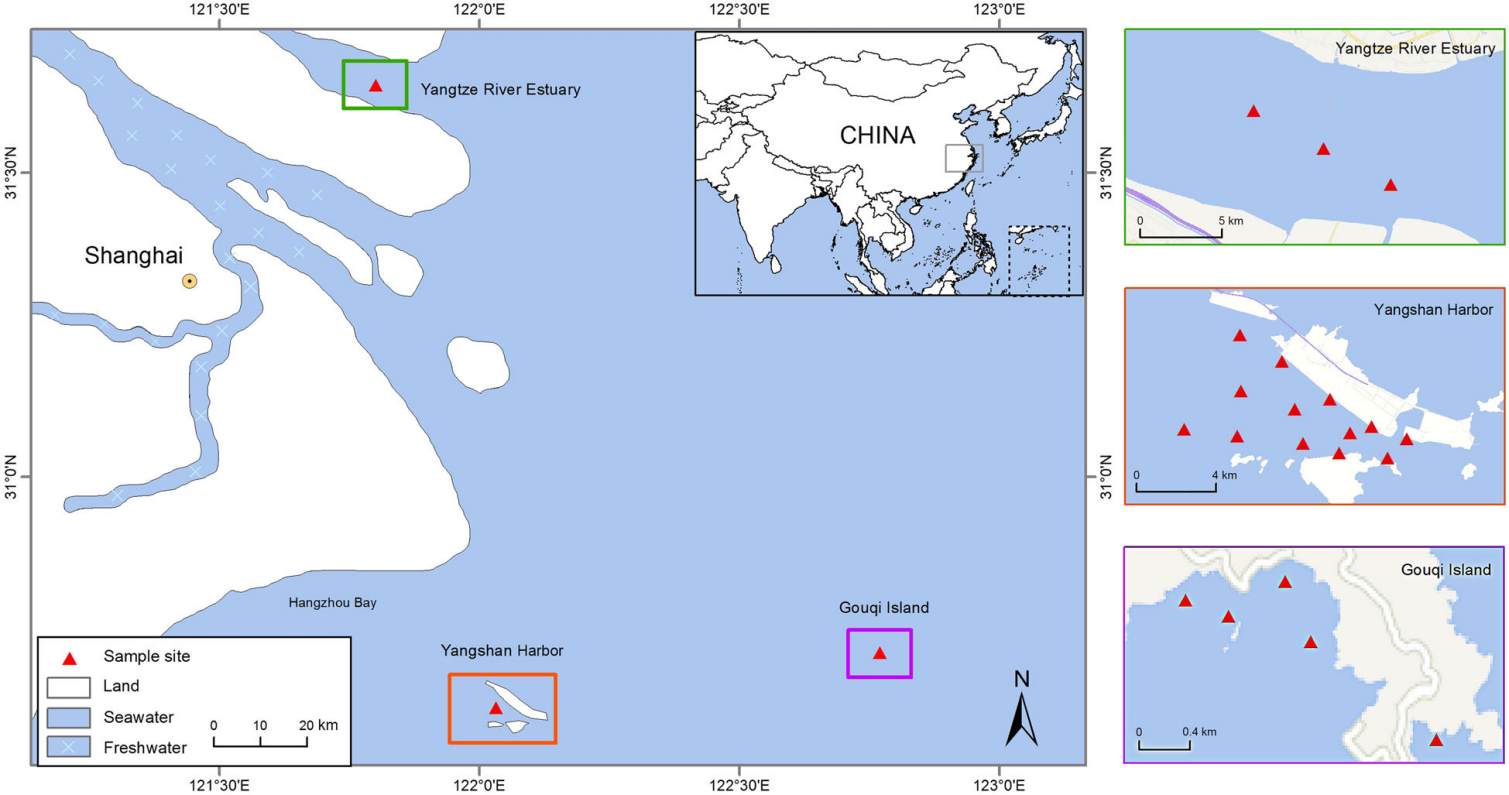
Schematic map of the sampling regions and sites in the East China Sea. Red triangles indicate the sampling sites where the waters were collected. YRE, Yangtze River Estuary. YSH, Yangshan Harbor. GQI, Gouqi Island. Map Source: Google Earth Pro, Mapabc.com, CNES / Astrium, TerraMetics. The maps were modified and integrated by using ArcGIS (Version 10.1, http://www.arcgis.com/index.html)

Approximately 200 L of surface seawater samples were taken from each of the three ECS regions: YSH, GQI, and YRE. The water samples were collected and mixed from 13 sampling sites in YSH, 5 sampling sites in GQI, and 3 sampling sites in YRE, taken at 3 different depths of 2 m, 5 m, and 8 m, for each sampling site (Fig. 8). The longitude and latitude of each sampling site are listed in Table S7. The temperature, salinity, total dissolved solid value, pH, and dissolved oxygen value of the water samples were measured (Table S8). The collected seawater was kept on ice and immediately transported to the laboratory for purification and concentration of viruses.

### Virus-like particles counting

Virus-like particles in surface seawater were enumerated using the protocols modified as described previously [58, 59]. Briefly, a single pooled sample representing each of the investigated ECS regions was processed for estimating viral abundance. Each pool consisted of 490 ml of water mixed from the different sites within each ECS region (Fig. 8). Pools were fixed with formaldehyde [2.0% (vol/vol) final concentration] on ice and transported to the laboratory. There, one milliliter of the formaldehyde-fixed water sample was immediately filtered through a 0.02 μm pore-size Anodisc membrane filter (Whatman). The filters were then stained with SYBR Green I (Invitrogen) and observed under an epifluorescence microscope (Axio Scope A1, Zeiss). The entire procedures for filtration and staining were done within 1 h. Each sample was randomly counted using ten microscopic fields [59].

### Virus isolation, DNA extraction, and sequencing

Viruses were purified and concentrated from water samples according to the procedures described in [60] and our previous work [61]. Approximately 200 L of water was subjected to viral isolation using 50 kDa tangential flow filtration followed by 30 kDa ultrafiltration. The obtained viral concentrates (approximately 5 ml) were then filtered through a 0.22 μm pore size filter and aliquoted for storage at − 80 °C. In order to remove environmental DNA, one μl (1 Unit) DNase I (Thermo Fisher) and 100 μl 10× reaction buffer (which contains MgCl_2_) were added into each aliquot of viral concentrate (approximately 1 ml) prior to the extraction of viral DNA, according to manufacturer’s instructions. Afterwards, viral DNA was extracted using the QIAGEN Blood and Tissue kit (QIAGEN). Approximately, 2.0 μg of viral DNA (2.1 μg for GQI, 2.0 μg for YSH, and 1.9 μg for YRE) was first disrupted with ultrasonic waves (Fisher Scientific). Fragments, ranging from 1000 to 1400 bp, were then purified with Agencout Ampure beads. Library construction was carried out using the GS DNA Library Preparation kit (Roche Applied Science), and sequencing (one run for each library) was performed on the Roche 454 Genome Sequencer FLX platform (Shanghai South Gene Technology co, Ltd). Meanwhile, the same amount of Milli-Q water was included as a negative control for DNA extraction. Because no DNA was extracted from the negative control, sequencing was aborted.

### Transmission electron microscopy

Viral morphotypes and purity (free of cell contamination) were determined using transmission electron microscopy. Briefly, one drop of the above purified and concentrated viral solution (approximately 10^12^ VLPs/ml) was loaded on a copper grid and dried at room temperature for 15 min. After excess liquid was drained off, the grid was stained with 2% phosphotungstic acid for 2 min. Excess phosphotungstic acid was removed and air-dried. The grids were then examined under a Philips TECNAI 12 transmission electron microscope at an acceleration voltage of 100 kV.

### Metagenomic sequence analysis

#### Quality control

Raw reads were analyzed by using Newbler v2.8 (454 Life Sciences) with the default parameters in order to trim off the adaptor and low-quality sequences. Reads with average quality scores lower than 25 and length shorter than 100 bp were removed. If reads were longer than 750 bp, the nucleotides that followed the 750 bp were trimmed. Cdhit-454 [62] was used to remove artificial duplicates with the default parameters.

#### Virome community analysis

After quality control, reads were firstly subjected to BLASTx analysis against the non-redundant (nr) protein sequences database downloaded through ftp://ftp.ncbi.nlm.nih.gov/blast/db/ on our local server, with the command line: blastx -query input.fasta -db nr -out output.result -evalue 1e-3 -outfmt 0. The MEGAN software (MEtaGenome Analyzer, version 5.7.1) [63] was used to assign taxonomic groups for viruses and cellular organisms (bacteria, archaea, and eukaryotes) to the virome reads with significant BLAST hits. The MEGAN-based taxonomic assignment was performed based on the top 10% of the significant hits.

Secondly, to avoid misclassifications caused by MEGAN [28], all clean reads from the three viromes were re-compared to a constructed local virus database by using BLASTx with the customized parameters: -evalue 1e-3 -outfmt 0. The local virus database was established with the command line: blastdb_alinstool -gilist txid10239ORGN.txt -db nr -out nr_viruses -title nr_viruses, which contained all the viral sequences retrieved from the nr database to our local server. Reads with significant matches were imported into the MEGAN software to assign taxonomic groups based on the top 10% of hits.

Thirdly, to avoid false positives caused by the size of the database (e-value of 0.001 is typically used against nr, but not necessarily small/local databases), the assigned viral reads (BLASTx search against the local virus database) from the viromes were searched (BLASTx, E-value 1e-3) against another local database. It contained all of the protein sequences predicted from both contigs that were longer than 5 kb and circular contigs in the GOV2 viral population datasets, containing 488,130 curated viral contigs. Additionally, the unknown reads that had no significant hits with nr were also compared to this viral protein database for further identification of their origin. The GOV2 datasets were downloaded from the iVirus database (https://datacommons.cyverse.org/browse/iplant/home/shared/iVirus/GOV2.0), and the open reading frames (ORFs) were predicted by using Prodigal [64] with the command line: prodigal -c -a final.contigs.orfs.faa -d final.contigs.orfs.fasta -i input.fasta -m -o final.contigs.txt -p meta –q.

#### Virus host analysis

Viruses in the water samples were identified by searching the constructed local virus database (see above) and then taxonomically assigned using the MEGAN software. Subsequently, the putative host information of the identified viral species was recorded by retrieving the host information of the matched viral species deposited in the NCBI taxonomy database with the scripts written in Python. The NCBI taxonomy database classifies the host of a virus as “archaea, bacteria, protozoa, algae, plants, fungi, invertebrates, vertebrates, or environment”. If the host of a virus could not be assigned, it was defined as unknown.

#### Analysis of the relative abundance of virus species in the viromes

The complete genomic sequences of the viruses were downloaded from the NCBI databases. They were then aligned to the viral metagenomic datasets obtained in this study using the tBLASTx program (E-value 1e-3) integrated in GAAS (Genome relative Abundance and Average Size software) [65] with default parameters (−f qualified_reads.fasta -d ref_viral_genome.fna –a txaids.txt -t virus_taxon.tre –v proteic). Reads with significant similarities (a minimum alignment identity ≥50, a minimum alignment relative length ≥ 50, an E-value ≤1e-3) were screened and weighted to a genome in order to determine the affiliation of taxonomy. Genome relative abundances were normalized by their size. The output files were subsequently analyzed to understand the relative abundance of diverse virus species in the viromes.

#### De novo assembly

Based on the BLASTx results, reads assigned to the local virus database, together with those assigned to unknown sequences based on the BLASTx search against the nr database, were retrieved and defined as virus-origin reads. The reads were then de novo assembled using the Geneious Pro version 7.06, with a minimum overlap of 25 bp and 95% of minimal match percentage. The assembled contigs and unused reads were retrieved as the result files.

#### ORF prediction and functional annotation

Viral ORFs from ECS viromes were predicted by using Prodigal [64] with the following command line: prodigal -a output_protein.fasta -d output_nucl.fasta -o genes.gff -s potential.stat –i input.fasta, and deduplicated using CD-HIT [66] with the command line: cd-hit -i input_protein.fasta -o output_protein.fasta -c 0.8 -n 5 -d 0. The ORFs longer than 100 amino acids were retained and subjected to annotation using the online version of eggNOG-mapper [67, 68] and then compared to the Conserved Domains Database (CDD) using the Batch CD-Search tool (data source: CDSEARCH/cdd v3.17, E-value 1e-2) [69].

#### Protospacers targeting analysis of viral metagenomes

A total of 720,391 CRISPR spacers from all CRISPR-Cas loci identified in the bacterial and archaeal genomes [70] were downloaded and used as queries to search against the assembled contigs and unused reads from the viromes by performing BLASTn with the customized parameters: -dust no -word_size 8 -evalue 1e-2 [70]. The filtered BLASTn hits with at least 95% identity and 95% coverage (only 1–2 mismatch(es) allowed) were considered to be protospacers [70]. The matched viral sequences were then compared to the established local virus database by BLASTx (E-value 1e-5), and BLASTx result files were imported into the MEGAN software to taxonomically assign each viral sequence.

#### Recruitment analysis of uncultured marine archaeal viruses in the ECS viromes

The sequences of an uncultured marine thaumarchaeal virus (38,209 bp, GenBank accession no. KR029602.1) [26] and 26 genomes of uncultured magroviruses (22–118 kbp) of the Marine Group II Euryarchaeota [27] were used as references to recruit reads from the ECS viromes (after quality control) by using Geneious with the parameter settings of minimum overlap length of 100 bp and minimum overlap identity of 90%.

## Supplementary information

**Additional file 1: Fig. S1.** Sequence length distribution of all contigs from the ECS viromes.

**Additional file 2: Fig. S2.** Contig_13 (the YSH virome) and 55 matched spacers from different *Listeria monocytogenes* isolates.

**Additional file 3: Fig. S3.** Read_IFVXWXA02D9OPB (the YSH virome) and 7 matched spacers from five *Klebsiella pneumoniae* and two *Pseudomonas aeruginosa* strains.

**Additional file 4: Fig. S4.** Read(s) mapping to sequence (contig_156409) of the Group A magrovirus (A) and the viral fosmid Oxic1_7 (B).

**Additional file 5: Table S1.** Genome coverage analysis of the top ten known viral species identified the ECS viromes.

**Additional file 6: Table S2.** Category of function class of predicted viral ORFs from the ECS viromes.

**Additional file 7: Table S3.** Function annotation using the Conserved Domains Database (CDD) of predicted viral ORFs from the ECS viromes.

**Additional file 8: Table S4.** Function annotation using the eggNOG-mapper of predicted viral ORFs from the ECS viromes.

**Additional file 9: Table S5.** Protospacers targeting analysis of the ECS viromes.

**Additional file 10: Table S6.** Spacers targeting sites.

**Additional file 11: Table S7.** Global Positioning System (GPS) of the sample sites.

**Additional file 12: Table S8.** Physical and chemical index values of the sampled water.

## Data Availability

The datasets generated and/or analyzed during the current study are available in the NCBI Sequence Read Archive (SRA) repository under the accession numbers of SRR7339788 (YSH), SRR7339789 (GQI), SRR7339790 (YRE). All data generated or analyzed during this study are included in this published article and its supplementary information files.

## Abbreviations

dsDNA: Double-stranded DNA
ECS: East China Sea
YRE: Yangtze River Estuary
YSH: Yangshan Harbor
GQI: Gouqi Island
ssDNA: Single-stranded DNA
GAAS: Genome relative Abundance and Average Size software
ORFs: Open reading frames
CDD: Conserved Domains Database
SRA: Sequence Read Archive
COG: Clusters of Orthologous Group
JRE: Jiulong River Estuary
VLPs: Virus-like particles
GOS: Global Ocean Sampling
CRISPR-Cas: Clustered regularly interspaced palindromic repeats and CRISPR associated proteins systems
MGE: Mobile genetic elements

## References

[1] Bergh O, Borsheim KY, Bratbak G, Heldal M (1989). High abundance of viruses found in aquatic environments. Nature.

[2] Breitbart M, Salamon P, Andresen B, Mahaffy JM, Segall AM, Mead D, Azam F, Rohwer F (2002). Genomic analysis of uncultured marine viral communities. Proc Natl Acad Sci U S A.

[3] Breitbart M, Felts B, Kelley S, Mahaffy JM, Nulton J, Salamon P, Rohwer F (2004). Diversity and population structure of a near-shore marine-sediment viral community. Proc Biol Sci.

[4] Angly FE, Felts B, Breitbart M, Salamon P, Edwards RA, Carlson C, Chan AM, Haynes M, Kelley S, Liu H (2006). The marine viromes of four oceanic regions. PLoS Biol.

[5] Ge X, Wu Y, Wang M, Wang J, Wu L, Yang X, Zhang Y, Shi Z (2013). Viral metagenomics analysis of planktonic viruses in East Lake, Wuhan, China. Virol Sin.

[6] Lopez-Bueno A, Tamames J, Velazquez D, Moya A, Quesada A, Alcami A (2009). High diversity of the viral community from an Antarctic lake. Science.

[7] Desnues C, Rodriguez-Brito B, Rayhawk S, Kelley S, Tran T, Haynes M, Liu H, Furlan M, Wegley L, Chau B (2008). Biodiversity and biogeography of phages in modern stromatolites and thrombolites. Nature.

[8] Yoshida M, Takaki Y, Eitoku M, Nunoura T, Takai K (2013). Metagenomic analysis of viral communities in (hado) pelagic sediments. PLoS One.

[9] Overbeek R, Begley T, Butler RM, Choudhuri JV, Chuang HY, Cohoon M, de Crecy-Lagard V, Diaz N, Disz T, Edwards R (2005). The subsystems approach to genome annotation and its use in the project to annotate 1000 genomes. Nucleic Acids Res.

[10] Overbeek R, Olson R, Pusch GD, Olsen GJ, Davis JJ, Disz T, Edwards RA, Gerdes S, Parrello B, Shukla M (2014). The SEED and the rapid annotation of microbial genomes using subsystems technology (RAST). Nucleic Acids Res.

[11] Benson DA, Cavanaugh M, Clark K, Karsch-Mizrachi I, Ostell J, Pruitt KD, Sayers EW (2018). GenBank. Nucleic Acids Res.

[12] Van Etten JL (2011). Another really, Really Big Virus. Viruses.

[13] Brum JR, Sullivan MB (2015). Rising to the challenge: accelerated pace of discovery transforms marine virology. Nat Rev Microbiol.

[14] Gong Z, Liang Y, Wang M, Jiang Y, Yang Q, Xia J, Zhou X, You S, Gao C, Wang J (2018). Viral diversity and its relationship with environmental factors at the surface and Deep Sea of Prydz Bay, Antarctica. Front Microbiol.

[15] Garin-Fernandez A, Pereira-Flores E, Glockner FO, Wichels A (2018). The North Sea goes viral: occurrence and distribution of North Sea bacteriophages. Mar Genomics.

[16] Taboada B, Isa P, Gutierrez-Escolano AL, Del Angel RM, Ludert JE, Vazquez N, Tapia-Palacios MA, Chavez P, Garrido E, Espinosa AC, et al. The Geographic Structure of Viruses in the Cuatro Cienegas Basin, a Unique Oasis in Northern Mexico, Reveals a Highly Diverse Population on a Small Geographic Scale. Appl Environ Microbiol. 2018;84(11). 10.1128/AEM.00465-18 PMC5960960 29625974

[17] Brum JR, Ignacio-Espinoza JC, Roux S, Doulcier G, Acinas SG, Alberti A, Chaffron S, Cruaud C, de Vargas C, Gasol JM (2015). Ocean plankton. Patterns and ecological drivers of ocean viral communities. Science (New York, NY).

[18] Cai L, Zhang R, He Y, Feng X, Jiao N. Metagenomic Analysis of Virioplankton of the Subtropical Jiulong River Estuary, China. Viruses. 2016;8(2). 10.3390/v8020035 PMC4776190 26848678

[19] Yan Q, Wang M, Bai X, Sun J, Liang Y, Wang F, Yang L, Liu G, Lu L (2010). New phylogenetically distinct cyanophages found in the coastal Yellow Sea by Qingdao. Acta Virol.

[20] Zheng Q, Jiao N, Zhang R, Wei J, Zhang F (2014). The evolutionary divergence of psbA gene in Synechococcus and their myoviruses in the East China Sea. PLoS One.

[21] Liang Y, Wang L, Wang Z, Zhao J, Yang Q, Wang M, Yang K, Zhang L, Jiao N, Zhang Y (2019). Metagenomic analysis of the diversity of DNA viruses in the surface and deep-sea of the South China Sea. Front Microbiol.

[22] Li W, Wang M, Pan H, Burgaud G, Liang S, Guo J, Luo T, Li Z, Zhang S, Cai L (2018). Highlighting patterns of fungal diversity and composition shaped by ocean currents using the East China Sea as a model. Mol Ecol.

[23] Wang H, Wu S, Li K, Pan Y, Yan S, Wang Y (2018). Metagenomic analysis of ssDNA viruses in surface seawater of Yangshan deep-Water Harbor, Shanghai, China. Mar Genomics.

[24] Roux S, Emerson JB, Eloe-Fadrosh EA, Sullivan MB (2017). Benchmarking viromics: an in silico evaluation of metagenome-enabled estimates of viral community composition and diversity. Peer J.

[25] Ondov BD, Bergman NH, Phillippy AM (2011). Interactive metagenomic visualization in a web browser. BMC Bioinformatics.

[26] Chow CE, Winget DM, White RA, Hallam SJ, Suttle CA (2015). Combining genomic sequencing methods to explore viral diversity and reveal potential virus-host interactions. Front Microbiol.

[27] Philosof A, Yutin N, Flores-Uribe J, Sharon I, Koonin EV, Beja O (2017). Novel abundant oceanic viruses of uncultured marine group II Euryarchaeota. Curr Biol.

[28] Bagci C, Beier S, Gorska A, Huson DH (2019). Introduction to the Analysis of Environmental Sequences: Metagenomics with MEGAN. Methods Mol Biol (Clifton, NJ).

[29] Sullivan MB, Lindell D, Lee JA, Thompson LR, Bielawski JP, Chisholm SW (2006). Prevalence and evolution of core photosystem II genes in marine cyanobacterial viruses and their hosts. PLoS Biol.

[30] Roux S, Krupovic M, Debroas D, Forterre P, Enault F (2013). Assessment of viral community functional potential from viral metagenomes may be hampered by contamination with cellular sequences. Open Biol.

[31] Koonin EV, Dolja VV, Krupovic M (2015). Origins and evolution of viruses of eukaryotes: the ultimate modularity. Virology.

[32] La Scola B, Desnues C, Pagnier I, Robert C, Barrassi L, Fournous G, Merchat M, Suzan-Monti M, Forterre P, Koonin E (2008). The virophage as a unique parasite of the giant mimivirus. Nature.

[33] Gong C, Zhang W, Zhou X, Wang H, Sun G, Xiao J, Pan Y, Yan S, Wang Y (2016). Novel Virophages discovered in a freshwater Lake in China. Front Microbiol.

[34] Zhou J, Sun D, Childers A, McDermott TR, Wang Y, Liles MR (2015). Three novel virophage genomes discovered from Yellowstone Lake metagenomes. J Virol.

[35] Zhou J, Zhang W, Yan S, Xiao J, Zhang Y, Li B, Pan Y, Wang Y (2013). Diversity of virophages in metagenomic data sets. J Virol.

[36] Labonte JM, Reid KE, Suttle CA (2009). Phylogenetic analysis indicates evolutionary diversity and environmental segregation of marine podovirus DNA polymerase gene sequences. Appl Environ Microbiol.

[37] Giovannoni SJ (2017). SAR11 Bacteria: the Most abundant plankton in the oceans. Annu Rev Mar Sci.

[38] Zhao X, Schwartz CL, Pierson J, Giovannoni SJ, McIntosh JR, Nicastro D. Three-Dimensional Structure of the Ultraoligotrophic Marine Bacterium “Candidatus Pelagibacter ubique”. Appl Environ Microbiol. 2017;83(3). 10.1128/AEM.02807-16 PMC5244296 27836840

[39] Pennekamp F, Pontarp M, Tabi A, Altermatt F, Alther R, Choffat Y, Fronhofer EA, Ganesanandamoorthy P, Garnier A, Griffiths JI (2018). Biodiversity increases and decreases ecosystem stability. Nature.

[40] Colombet J, Billard H, Viguès B, Balor S, Boulé C, Geay L, Benzerara K, Menguy N, Ilango G, Fuster M (2019). Discovery of high abundances of aster-like nanoparticles in pelagic environments: characterization and dynamics. Front Microbiol.

[41] Prangishvili D, Bamford DH, Forterre P, Iranzo J, Koonin EV, Krupovic M (2017). The enigmatic archaeal virosphere. Nat Rev Microbiol.

[42] Abergel C, Legendre M, Claverie JM (2015). The rapidly expanding universe of giant viruses: Mimivirus, Pandoravirus, Pithovirus and Mollivirus. FEMS Microbiol Rev.

[43] Legendre M, Lartigue A, Bertaux L, Jeudy S, Bartoli J, Lescot M, Alempic JM, Ramus C, Bruley C, Labadie K (2015). In-depth study of Mollivirus sibericum, a new 30,000-y-old giant virus infecting Acanthamoeba. Proc Natl Acad Sci U S A.

[44] Legendre M, Bartoli J, Shmakova L, Jeudy S, Labadie K, Adrait A, Lescot M, Poirot O, Bertaux L, Bruley C (2014). Thirty-thousand-year-old distant relative of giant icosahedral DNA viruses with a pandoravirus morphology. Proc Natl Acad Sci U S A.

[45] Hwang J, Park SY, Lee S, Lee TK (2018). High diversity and potential translocation of DNA viruses in ballast water. Mar Pollut Bull.

[46] Koonin EV, Makarova KS (2019). Origins and evolution of CRISPR-Cas systems. Philos Trans R Soc Lond Ser B Biol Sci.

[47] Zhang J, Kasciukovic T, White MF (2012). The CRISPR associated protein Cas4 is a 5′ to 3′ DNA exonuclease with an iron-sulfur cluster. PLoS One.

[48] Amitai G, Sorek R (2016). CRISPR-Cas adaptation: insights into the mechanism of action. Nat Rev Microbiol.

[49] Hille F, Richter H, Wong SP, Bratovic M, Ressel S, Charpentier E (2018). The biology of CRISPR-Cas: backward and forward. Cell.

[50] Sorek R, Lawrence CM, Wiedenheft B (2013). CRISPR-mediated adaptive immune systems in bacteria and archaea. Annu Rev Biochem.

[51] Edwards RA, McNair K, Faust K, Raes J, Dutilh BE (2016). Computational approaches to predict bacteriophage-host relationships. FEMS Microbiol Rev.

[52] Ramaswamy V, Cresence VM, Rejitha JS, Lekshmi MU, Dharsana KS, Prasad SP, Vijila HM (2007). Listeria--review of epidemiology and pathogenesis. J Microbiol Immunol Infect.

[53] Razin A, Sedat JW, Sinsheimer RL (1973). In vivo methylation of replicating bacteriophage phi chi174 DNA. J Mol Biol.

[54] Magrini V, Salmi D, Thomas D, Herbert SK, Hartzell PL, Youderian P (1997). Temperate Myxococcus xanthus phage Mx8 encodes a DNA adenine methylase, Mox. J Bacteriol.

[55] Mendoza SD, Nieweglowska ES, Govindarajan S, Leon LM, Berry JD, Tiwari A, Chaikeeratisak V, Pogliano J, Agard DA, Bondy-Denomy J (2020). A bacteriophage nucleus-like compartment shields DNA from CRISPR nucleases. Nature.

[56] Koonin EV, Krupovic M (2020). Phages build anti-defence barriers. Nat Microbiol.

[57] Roux S, Brum JR, Dutilh BE, Sunagawa S, Duhaime MB, Loy A, Poulos BT, Solonenko N, Lara E, Poulain J (2016). Ecogenomics and potential biogeochemical impacts of globally abundant ocean viruses. Nature.

[58] Wen K, Ortmann AC, Suttle CA (2004). Accurate estimation of viral abundance by epifluorescence microscopy. Appl Environ Microbiol.

[59] Patel A, Noble RT, Steele JA, Schwalbach MS, Hewson I, Fuhrman JA (2007). Virus and prokaryote enumeration from planktonic aquatic environments by epifluorescence microscopy with SYBR green I. Nat Protoc.

[60] Thurber RV, Haynes M, Breitbart M, Wegley L, Rohwer F (2009). Laboratory procedures to generate viral metagenomes. Nat Protoc.

[61] Sun G, Xiao J, Wang H, Gong C, Pan Y, Yan S, Wang Y (2014). Efficient purification and concentration of viruses from a large body of high turbidity seawater. MethodsX.

[62] Niu B, Fu L, Sun S, Li W (2010). Artificial and natural duplicates in pyrosequencing reads of metagenomic data. BMC bioinformatics.

[63] Huson DH, Auch AF, Qi J, Schuster SC (2007). MEGAN analysis of metagenomic data. Genome Res.

[64] Hyatt D, Chen G-L, LoCascio PF, Land ML, Larimer FW, Hauser LJ (2010). Prodigal: prokaryotic gene recognition and translation initiation site identification. BMC Bioinformatics.

[65] Angly FE, Willner D, Prieto-Davo A, Edwards RA, Schmieder R, Vega-Thurber R, Antonopoulos DA, Barott K, Cottrell MT, Desnues C (2009). The GAAS metagenomic tool and its estimations of viral and microbial average genome size in four major biomes. PLoS Comput Biol.

[66] Li W, Godzik A (2006). Cd-hit: a fast program for clustering and comparing large sets of protein or nucleotide sequences. Bioinformatics.

[67] Huerta-Cepas J, Forslund K, Coelho LP, Szklarczyk D, Jensen LJ, von Mering C, Bork P (2017). Fast genome-wide functional annotation through Orthology assignment by eggNOG-mapper. Mol Biol Evol.

[68] Huerta-Cepas J, Szklarczyk D, Forslund K, Cook H, Heller D, Walter MC, Rattei T, Mende DR, Sunagawa S, Kuhn M (2016). eggNOG 4.5: a hierarchical orthology framework with improved functional annotations for eukaryotic, prokaryotic and viral sequences. Nucleic Acids Res.

[69] Marchler-Bauer A, Bo Y, Han L, He J, Lanczycki CJ, Lu S, Chitsaz F, Derbyshire MK, Geer RC, Gonzales NR (2017). CDD/SPARCLE: functional classification of proteins via subfamily domain architectures. Nucleic Acids Res.

[70] Shmakov SA, Sitnik V, Makarova KS, Wolf YI, Severinov KV, Koonin EV. The CRISPR Spacer Space Is Dominated by Sequences from Species-Specific Mobilomes. MBio. 2017;8(5). 10.1128/mBio.01397-17 PMC5605939 28928211

