# Supplementary figures and images for: Diverse and unique viruses discovered in the surface water of the East China Sea
Source: BMC Genomics. 2020 Jun 26;21:441. doi: 10.1186/s12864-020-06861-y (PMC7318539; doi:10.1186/s12864-020-06861-y)

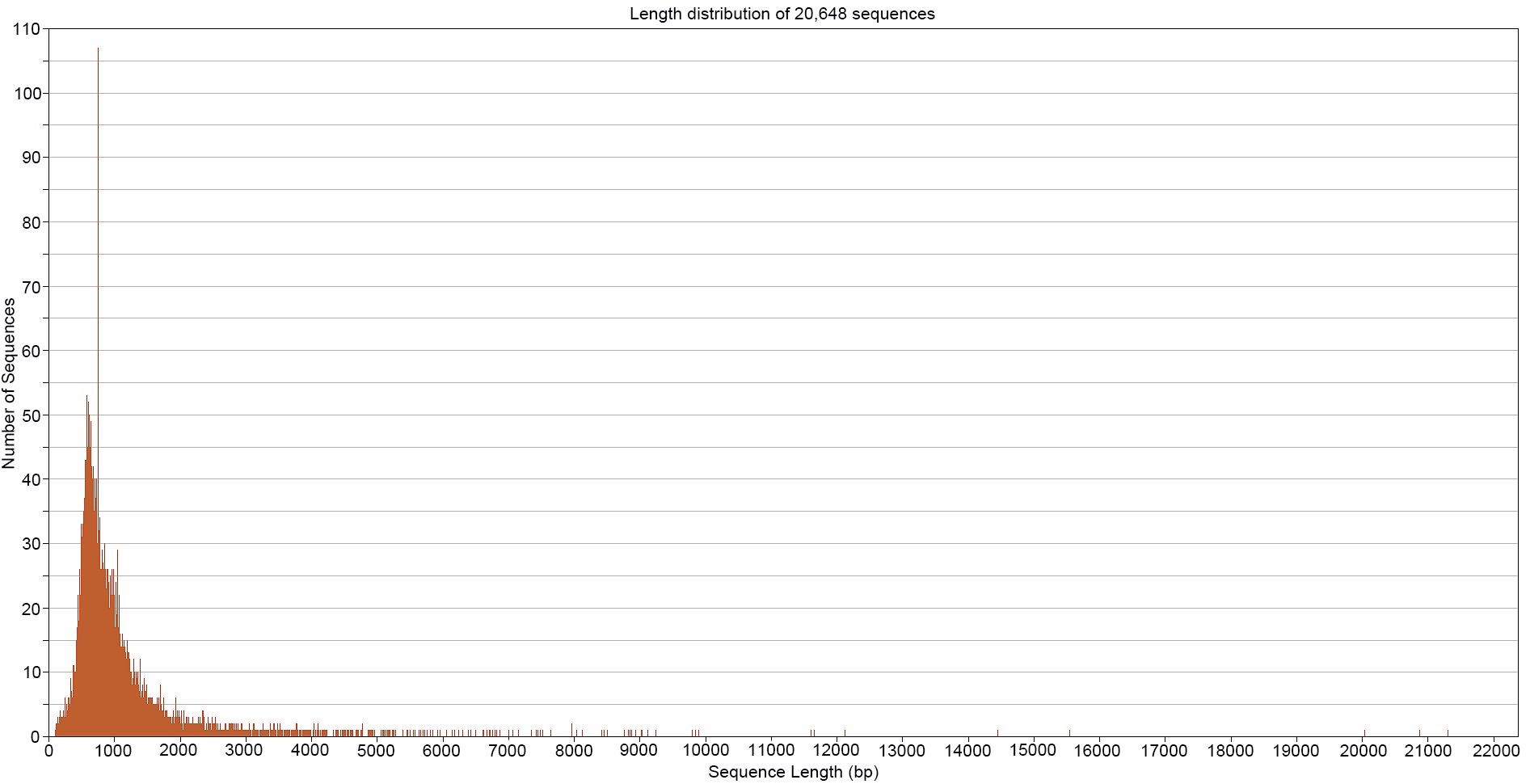

Supplement: Supplementary file 1 — Additional file 1: Fig. S1. Sequence length distribution of all contigs from the ECS viromes. [file 12864_2020_6861_MOESM1_ESM.png]

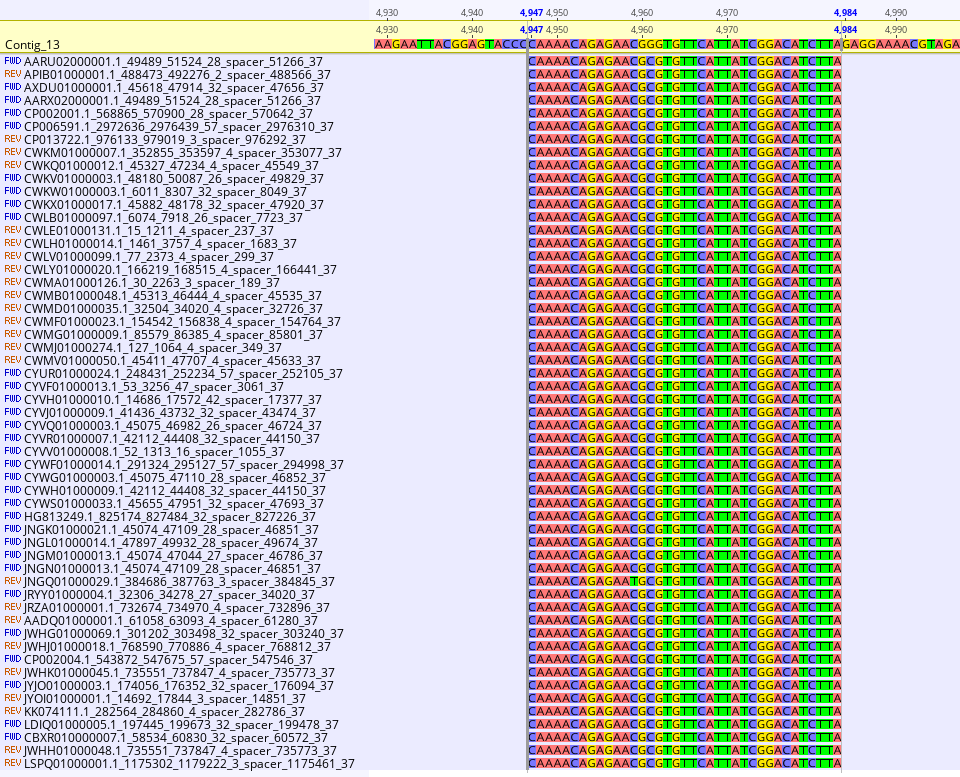

Supplement: Supplementary file 2 — Additional file 2: Fig. S2. Contig_13 (the YSH virome) and 55 matched spacers from different Listeria monocytogenes isolates. [file 12864_2020_6861_MOESM2_ESM.png]

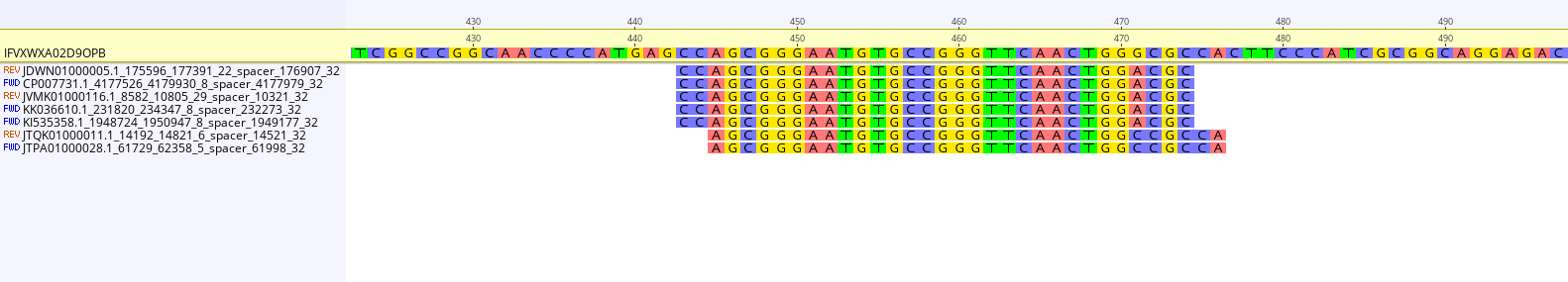

Supplement: Supplementary file 3 — Additional file 3: Fig. S3. Read_IFVXWXA02D9OPB (the YSH virome) and 7 matched spacers from five Klebsiella pneumoniae and two Pseudomonas aeruginosa strains. [file 12864_2020_6861_MOESM3_ESM.png]

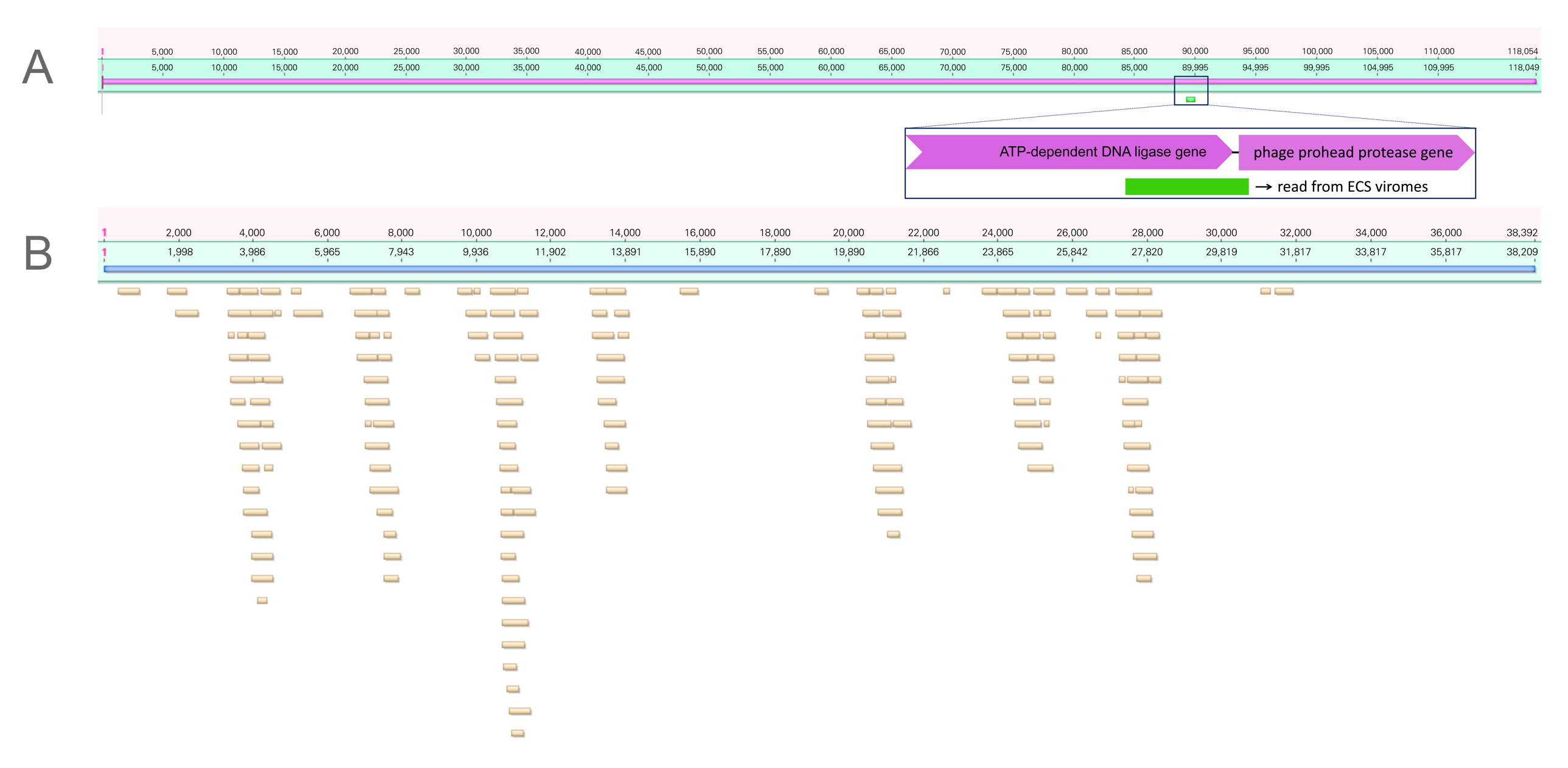

Supplement: Supplementary file 4 — Additional file 4: Fig. S4. Read(s) mapping to sequence (contig_156409) of the Group A magrovirus (A) and the viral fosmid Oxic1_7 (B). [file 12864_2020_6861_MOESM4_ESM.jpg]
